# Prevalence of patellar chondropathy on 3.0 T magnetic resonance imaging

**DOI:** 10.1590/0100-3984.2019.0105

**Published:** 2020

**Authors:** Eduardo André Gomes Krieger, Francisco Consoli Karam, Ricardo Bernardi Soder, Jefferson Luis Braga da Silva

**Affiliations:** 1 Hospital São Lucas da Pontifícia Universidade Católica do Rio Grande do Sul (PUCRS), Porto Alegre, RS, Brazil.

**Keywords:** Chondromalacia patellae, Chondropathy, Magnetic resonance imaging, Condromalácia da patela, Condropatias, Ressonância magnética

## Abstract

**Objective:**

To establish the prevalence of patellar chondropathy using 3T magnetic resonance imaging (MRI) and to correlate the findings with individual features such as gender, age, and body mass index.

**Materials and Methods:**

Data consisted of collecting 3T MRIs of patients’ knees obtained between October 2016 and September 2017, comprising a period of 12 months. These MRIs were assessed by an experienced musculoskeletal radiologist who confirmed the presence of patellar chondropathy and, when present, rated the finding into the four grades ascribed by the International Cartilage Repair Society.

**Results:**

A total number of 291 patients were assessed during the period with 389 MRI scans. Of those patients, 308 (79.2%) were diagnosed with patellar chondropathy, while 81 (20.8%) were not. Chondropathy was more prevalent in the female gender, in subjects above 40 years of age, and in obese patients. When the results were weighed in International Cartilage Repair Society classification, the milder grades (1 and 2) were seen in younger men (< 30 years of age), while the more severe grades (3 and 4) were mostly present in females, those above 40 years of age, and in obese patients.

**Conclusion:**

There was a high prevalence of patellar chondropathy in patients who had undergone high-field knee MRIs (79.2%), being highest in the female gender and in subjects above 40 years of age. The most prevalent group was graded as 4 by the International Cartilage Repair Society classification.

## Abstract (pt)

**Objetivo:**

Estabelecer a prevalência de condropatia patelar em exames de ressonância magnética (RM) em campo de 3,0 T e associar os achados com características como gênero, idade e índice de massa corporal.

**Materiais e Métodos:**

Foram coletados exames de RM de joelho em campo magnético de 3.0 T no período de outubro de 2016 a setembro de 2017. Os exames foram analisados por radiologista com experiência em radiologia musculoesquelética, que verificou a presença de condropatia patelar e a classificou entre os quatro graus, de acordo com a classificação da International Cartilage Repair Society.

**Resultados:**

Foram avaliados 291 pacientes, com a realização de 389 exames de RM. Destes exames, 308 (79,2%) apresentavam condropatia patelar e apenas 81 (20,8%) não a apresentavam. A doença foi vista mais frequentemente nas mulheres, em indivíduos acima de 40 anos e em obesos. Quando classificada por graus, os mais leves (graus 1 e 2) foram mais observados em homens e jovens (< 30 anos) e os mais severos (graus 3 e 4) no sexo feminino, nos acima de 40 anos e nos obesos.

**Conclusão:**

A prevalência de condropatia patelar nos pacientes que realizaram RM foi elevada (79,2%), sendo maior no sexo feminino e nos indivíduos acima de 40 anos. Dentro da classificação da International Cartilage Repair Society, o grupo mais prevalente foi o grau 4.

## INTRODUCTION

Patellar chondropathy is characterized by abnormal damage to the articular cartilage of the patella that can cause pain, especially in the anterior region of the knee. This damage can destroy cartilage integrity or even result in loss of substance, leading to exposure of the subchondral bone which is irreversible. Epidemiologically, change in the patellar cartilage was observed in 40% to 60% of patients at autopsies and in 20% to 50% of patients during arthrotomy for another diagnosis^(1)^.

The clinical picture is characterized by diffuse pain in the anterior region of the knee, mainly when squatting, going up and down stairs, or when the patient remains for long periods with the joint flexed, which is known as “cinema sign”.

The gold standard method for the diagnosis of the disease is arthroscopy and is revealed by a “softening” of the cartilage leading to the exposure of the subchondral bone. However, this procedure is not indicated for diagnosis since, if no treatable chondral lesions are found, it becomes an overly expensive diagnostic method, causes short-term functional limitation, pain, and stress, and exposes the patient to anesthetic and surgical risks^(2)^. MRI can diagnose patellar chondropathy and is currently considered the imaging test of choice since it is a non-invasive method with a complication rate lower than that of diagnostic arthroscopy. MRI sensitivity for chondral lesions varies between 57-86%, specificity varies between 74-93%, and the accuracy of diagnosis varies between 73-90%^(3-5)^.

Current studies are generally conducted on 1.5 tesla (T) devices, as they are the devices most commonly found in clinical practice. The 3T devices have become increasingly widespread due to their higher spatial resolution and thinner cuts than older devices. Although these 3T devices have shown greater accuracy in the diagnosis of patellar chondropathy^(6)^, further studies are necessary to establish their prevalence.

This study aimed at establishing the prevalence of patellar chondropathy in patients being examined by MRI at an extremely high magnetic field (3T). It also aimed at determining the relationship between the demographic and anthropometric variables of patients, such as gender, age, and body mass index (BMI), and classifying the severity of chondropathy, associating it with these variables.

## MATERIALS AND METHODS

The research was submitted and approved by the ethics committee of the institution where the study was conducted. The sample was calculated using WinPEPI version 11.43 software. Using a 95% confidence level, a 5% error margin, and an estimated prevalence of patellar chondropathy between 50-70%, the software indicated a minimum requirement of 385 examinations.

**Figure 1:**
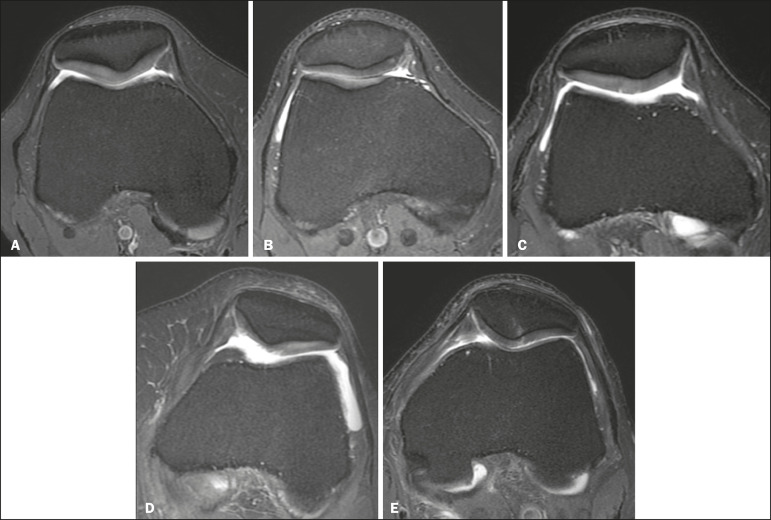
MR images according to the ICRS grades: normal examination (**A**), grade 1 (**B**), grade 2 (**C**), grade 3 (**D**), and grade 4 (**E**).

The knee MRI examinations were collected between October 2016 and September 2017 in a high magnetic field device (3T) and were classified by an experienced radiologist in the musculoskeletal system. The classification considered the presence or absence of patellar chondropathy and, when present, the radiologist classified it according to the International Cartilage Repair Society (ICRS) (Table 1 and Figure 1). The radiologist had no prior access to patient data. Examinations that did not allow evaluation of the images (for example, artifacts or other changes in the images) and patients under the age of 18 were excluded.

**Table 1:** Classification according to ICRS.

Grade	Arthroscopy	MRI
1	Softening of the cartilage. It may show superficial lesions	Hypersignal foci with normal contours
2	Cartilage fraying. Lesions covering less than 50% of the articular surface	Fissures in articular cartilage
3	Substantial loss of articular cartilage representing more than 50% of the thickness of the articular surface	Partial loss of cartilage thickness with focal ulceration
4	Complete loss of articular cartilage, with exposure of the subchondral bone	Total loss of chondral thickness with bone reaction

Data such as gender, age, weight, and height were reported by the patients at the time of the examination. BMI was calculated based on the patient’s height and weight, with the patient being classified (according to World Health Organization guidelines) as: 18.5, below ideal weight; 18.5 to 24.9, normal weight; from 25 to 29.9, overweight; equal to or above 30, obese^(7)^.

The examinations were performed using a 3T MRI device (Signa; GE Medical Systems, Waukesha, WI, USA) and a dedicated knee coil with eight channels. The examination was performed following four sequences; two with fat-suppressed T2-weighted fast spin-echo in the sagittal and coronal planes, one with proton density-weighted fat suppression in the axial plane, and the other with T1-weighted spin-echo in the sagittal plane. The parameters used for image acquisition were a 352 × 320 matrix, a field of view of 16 cm, and slice thickness of 3.03 cm with a 0.3 mm gap between slices.

The data were entered into a Microsoft Excel spreadsheet and analyzed using the SPSS statistical package version 21.0 (IBM Corp., Armonk, NY, USA). The normality of data distribution was tested with the Kolmogorov-Smirnov test. Continuous variables were described using measures of central tendency (mean) and dispersion (standard deviation). Categorical variables were represented by absolute and relative frequency. The association between categorical variables was determined using Pearson’s chi-square tests, together with the analysis of adjusted residuals. A value of *p* <0.05 was considered significant.

## RESULTS

Two hundred and ninety-one patients were evaluated, and the results of 389 MRI examinations of the knee were analyzed. Of the total number of MRI scans, 216 (55.5%) were conducted on females, and 173 (44.5%) on males. The mean age of the patients was 46.3 ± 15.3 years (ranging between 18 and 86 years), with a predominance of normal weight (n = 148; 38%) and overweight (n = 148; 38%) individuals. Ninety-three individuals were obese (23.9%). A total of 79.2% (n = 308) of the examinations showed indication of patellar chondropathy, whereas 20.8% (n = 81) did not.

The analysis of results by gender showed the highest rate of patellar chondropathy in females, with 88% being affected, and 68.2% of males being affected (*p* < 0.001). The disease was more prevalent in patients over 50 years of age, being detected in 95.1% of the examinations of individuals aged 50 to 59 years and 94.1% of those over 60 years, contrasting with the prevalence of 56.1% found in patients under 30 years old.

Regardless of age, the results on the nutritional status of patients, showed that those under ideal weight, with a BMI < 18.5, had a low prevalence. The other categories-normal weight, overweight, and obese-had a high prevalence on MRI of changes in patellar cartilage; 77.7% in normal-weight individuals, 77.2% in overweight individuals, and 86% in obese individuals. These results are detailed in Table 2.

**Table 2:** Presence or absence of patellar chondropathy by gender, age group, and nutritional status.

Variables	Patellar chondropathy			
	N	Without	With	*P*
Gender				< 0.001
Male	173	55 (31.8%)	118 (68.2%)	
Female	216	26 (12.0%)	190 (88.0%)	
Age group (years)				< 0.001
< 30	66	37 (56.1%) *	29 (43.9%)	
30 to 39	76	24 (31.6%) *	52 (68.4%)	
40 to 49	81	11 (13.6%)	70 (86.4%)	
50 to 59	81	4 (4.9%)	77 (95.1%) *	
≥ 60	85	5 (5.9%)	80 (94.1%) *	
Nutritional status				0.07
Underweight	3	2 (66.7%)	1 (33.3%)	
Normal weight	148	33 (22.3%)	115 (77.7%)	
Overweight	145	33 (22.8%)	112 (77.2%)	
Obese	93	13 (14.0%)	80 (86.0%)	

* Statistically significant association by analysis of adjusted residuals at a 5% significance level.

When our findings were classified according to the ICRS, they showed a prevalence of grade 4 in 29.3% (n = 114) of the examinations, grade 3 in 20.8% (n = 81), grade 2 in 14.9% (n = 58), grade 1 in 14,1% (n = 55), and no patellar chondropathy in 20.8% (n = 81) of the examinations.

Table 3 shows that most of the 173 examinations performed on male patients showed findings of grade 4 patellar chondropathy (n = 38; 22%). Of the 216 tests performed on women, in turn, 76 (35.2%) were classified as grade 4 patellar chondropathy.

**Table 3:** Distribution of the degree of chondropathy by gender, age group, and nutritional status

Variables	N	Without	Degree of patellar chondropathy				*P*
			Grade 1	Grade 2	Grade 3	Grade 4	
Gender							< 0.001
Male	173	55 (31.8%) *	20 (11.6%)	34 (19.7%) *	26 (15.0%)	38 (22.0%)	
Female	216	26 (12.0%)	35 (16.2%)	24 (11.1%)	55 (25.5%) *	76 (35.2%) *	
Age group (years)							< 0.001
< 30	66	37 (56.1%) *	9 (13.6%)	8 (12.1%)	8 (12.1%)	4 (6.1%)	
30 to 39	76	24 (31.6%) *	16 (21.1%)	8 (10.5%)	18 (23.7%)	10 (13.2%)	
40 to 49	81	11 (13.6%)	10 (12.3%)	14 (17.3%)	16 (19.8%)	30 (37.0%)	
50 to 59	81	4 (4.9%)	9 (11.1%)	14 (17.3%)	20 (24.7%)	34 (42.0%) *	
≥ 60	85	5 (5.9%)	11 (12.9%)	14 (16.5%)	19 (22.4%)	36 (31.6%) *	
Nutritional status							< 0.001
Underweight	3	2 (66.7%) *	0 (0.0%)	0 (0.0%)	0 (0.0%)	1 (33.3%)	
Normal weight	148	33 (22.3%)	28 (18.9%) *	17 (11.5%)	34 (23.0%)	36 (24.3%)	
Overweight	145	33 (22.8%)	18 (12.4%)	35 (24.1%) *	22 (15.2%)	37 (25.5%)	
Obese	93	13 (14.0%)	9 (9.7%)	6 (6.5%)	25 (26.9%)	40 (43.0%) *	

* Statistically significant association by analysis of adjusted residuals at a 5% significance level.

In patients under 30 years of age, 56.1% of the examinations (n = 37) showed the absence of patellar chondropathy, this being the most common finding. On the other hand, the finding of grade 4 patellar chondropathy was the least common, accounting for only 6.1% (n = 4) of the examinations. In those aged 30 to 39, the most common finding was also the absence of patellar chondropathy, comprising 31.6% of the examinations (n = 24), and the least common finding was grade 2 patellar chondropathy, with only 8 patients (10.5%). In patients over 40 years of age, grade 4 patellar chondropathy was the most common finding, accounting for 37% of the examinations (n = 30) and grade 1 patellar chondropathy the least common, with 10 knees (12.3%). In patients aged 50 to 59 years, the disease in its grade 4 was observed in 42% of the series of images (n = 34) and absent in findings of only 4 examinations (4.9%). This same pattern was observed in those over 60 years of age, with 31.6% of the examinations (n = 36) having some severe chondral injury and 5 (5.9%) not.

In underweight individuals (BMI < 18.5 kg/m^2^), the prevalence of patellar chondropathy on MRI (33.3%; n = 1) was lower than its absence (66.7%; n = 2). In the 148 normal-weight individuals, grade 3 was the most prevalent grade of the pathology, accounting for 23% (n = 34) of the total, closely followed by the group with no changes in patellar cartilage (22.3%; n = 33). In overweight individuals, who are characterized by a BMI of 25 to 29.9 kg/m^2^, the most common findings were grade 2 patellar chondropathy (24.1%; n = 35) and absence of signs (22.8%; n = 33). When assessing overweight individuals, for those with a BMI greater than or equal to 30 kg/m^2^, most MR images were classified as grade 4, comprising 43% (n = 40) of this population, while the less prevalent classifications were grade 1 and 2, with 9.7% (n = 9) and 6.5% (n = 6), respectively. These data are shown in Table 3.

## DISCUSSION

The patella is the site with the highest incidence of pain in the anterior region of the knee. It is the area where the degeneration process of this joint begins, especially after 40 years of age^(8)^. Widuchowski et al. also found that the patella is the site with the highest prevalence of chondral lesions compared to femoral condyles and tibial plateaus^(9)^.

The findings of the present study showed a high prevalence of patellar chondropathy in MRI examinations performed using a device with an extremely high magnetic field (3T), reaching 79.2% of the cases. This high value is greater than that found in other diagnostic methods such as macroscopy, which reports a prevalence between 40-60%^(1)^, and examinations using the same method but with devices with a smaller field, such as those using 1.5T devices^(10,11)^. This indicates that the development of technology will allow an increase in the sensitivity and specificity of the examination, especially in the early stages of the disease, as a result of examinations with a higher magnetic field and the use of specific software to map articular cartilage, such as T2 mapping^(12)^. Despite this, we cannot say that one method is superior to the other since we did not compare it to the gold standard method, arthroscopy.

Patellar chondropathy was more prevalent in females (88%) than in males (68.2%). These findings corroborate the previous literature, which reports that women have a higher risk of damage to the articular cartilage of the patella^(13)^.

Chondral lesions are known to occur not only on the patella but also on the femoral condyles and tibial plateaus which, in older patients, are signs of knee osteoarthrosis^(13,14)^. Our results corroborate this finding. The percentage of patients over 50 years old with patellar chondropathy was significant (95.1% in patients aged between 50 and 59 years, and 94.1% in those over 60 years of age). It is noteworthy that the prevalence was high even in patients below 30 years old, accounting for around 45%, and surprisingly high, from 30 to 40 years old, reaching almost 70%. These data indicate that the knee joint begins to age early and that it is necessary to seek the causes of this problem.

Another important risk factor for the development of chondral patellar injuries is weight, which in this work is expressed by BMI. In our sample, the highest percentage of injuries was observed in individuals with a BMI ≥ 30 kg/m^2^, according to the literature^(13-15)^.

In our study, most of the examinations were classified as grade 4 patellar chondropathy, which is inconsistent with Widuchowski et al.^(9)^, who indicated grade 2 chondral injury as the most frequent grade, and Curl et al., who indicated grade 3 as the most frequent^(8)^. However, the classification used in these studies was based on arthroscopic findings rather than MRI. Research using MRI findings for classification, divides the four grades of patellar chondropathy into two new categories: initial chondropathy, covering grades 1 and 2, and severe chondropathy, covering grades 3 and 4. Generally, this division is considered because there are subtle differences at the time of radiological classification^(16,17)^. Grade 4 chondropathy was unexpectedly the most prevalent form of the pathology in patients over 40 years of age. Although most of the patients under the age of 40 did not have chondropathy, there was prevalence of severe chondropathy in the elderly, indicating that it evolves rapidly from an established chondropathy. This is not discussed in the literature.

It should be noted that classifying chondropathy is the subject of much controversy. The terms chondromalacia and chondropathy are already often confusing. Outerbridge used the term chondromalacia, which theoretically is the first stage of patellar chondropathy, when describing and classifying the pathology. Interestingly, even publications in recent years have continued using the term chondromalacia to refer to all grades of the disease^(16,18)^. In 2005, Grelsamer asked what term would replace the term chondromalacia, since the softening of the cartilage could not be used to denote the different stages of the disease^(19)^. We agree with this author and suggest the use of the term “patellar chondropathy”, as it is a disease of the cartilage of the patella that covers various degrees of impairment.

The limitations of this study include the classification of only one radiologist, which could cause bias when classifying according to the ICRS. However, this study was not primarily aimed at assessing the interobserver and intraobserver agreement of readers but to categorize the patients regarding whether or not they have patellar chondropathy. There are obvious criteria for identifying chondropathy in imaging exams. Another limitation is that this is a retrospective study using MRIs obtained from patients who were indicated for undergoing the procedure. Therefore, we cannot extrapolate these prevalence findings to the population. For that, we would have to perform MRI on patients’ knees on a representative sample of the population. In addition to these limitations, when studying patellar cartilage by MRI, it is possible to use a specific coil, sequence protocol, and software improving diagnostic accuracy, such as T2 mapping^(12)^. In the present study, the protocol used was not designed for this purpose. Finally, the ideal scenario would be to compare the results with that obtained with the gold standard for diagnosis, arthroscopy, but due to economic reasons and because this is a retrospective study with data collected from a database, it was not possible to make this comparison.

## CONCLUSION

The prevalence (79.2%) of patellar chondropathy was high in patients who underwent knee MRI using a 3T device. The prevalence of patellar chondropathy was higher in females and the elderly (over 40 years of age). No difference was found in the prevalence of chondropathy in normal-weight, overweight, and obese patients. Most of the diagnosed patients were identified as having patellar chondropathy grade 4.
